# Family Life Courses, Uncertain Futures, and the Changing World of Work: State-of-the-Art and Prospects

**DOI:** 10.1007/s10680-024-09701-x

**Published:** 2024-05-30

**Authors:** Anna Matysiak, Daniele Vignoli

**Affiliations:** 1 Interdisciplinary Center for Labour Market and Family Dynamics (LabFam), Faculty of Economic Sciences, University of Warsaw, Ul. Długa 44/50, 00-241 Warsaw, Poland; 2 Department of Statistics, Computer Science, Applications, University of Florence, Viale G.B. Morgagni 59, 50134 Florence, Italy

## Abstract

Labour markets in post-industrial countries have been undergoing tremendous transformations in the last two decades, substantially changing the conditions in which young adults take family decisions and raise children. Whilst these changes create new opportunities, they also generate risks which potentially foster uncertain futures and affect individuals’ opportunities to earn income, provide care for family members, and make long-term commitments. This Special Issue aims to stimulate the debate on the effects of rapid labour market transformations and growing uncertainty on families in contemporary wealthiest countries. Its articles suggest that economic uncertainty, the threat of unemployment or precarious employment, and financial difficulties lead to fertility postponement and increase the risk of union disruption. These effects intensify when labour market deregulation goes in tandem with labour market dualization and become more pronounced during periods of economic hardship, such as economic recessions or the COVID-19 pandemic. In addition, the effects of economic activity on family-related behaviours have become less gendered as women increasingly gain economic independence. Finally, it appears that highly educated workers and members of the upper social classes face increasingly better conditions for realising their fertility intentions than their lower-educated counterparts and those of the lower social classes. In this introductory article, we review the theoretical premises and the empirical evidence to provide a comprehensive background on what labour force participation and its conditions imply for family life courses. We then introduce the articles collected in this Special Issue and conclude with a discussion on prospects for future research.

## Introduction

In post-industrial countries, labour markets have been undergoing tremendous transformations over the past two decades, substantially changing the conditions in which young adults take family decisions and raise children. These changes are largely driven by the ongoing integration of the global economy, rapid technological change, and the upcoming shift towards a green economy. They create new opportunities for workers, but also generate risks, such as potentially fostering uncertain futures and affecting individuals’ chances to earn income, provide care for family members, and make long-term commitments.

On the positive side, wealthy societies have experienced advances in information and communication technologies (ICT), significant decreases in transportation costs, and increased purchasing power. New jobs are being created in rapidly expanding sectors. These are largely information and communication sectors, as well high-tech industries, but also care (including healthcare and long-term care) and small service economy jobs (e.g. cleaning, delivery) (World Bank, 2018). Opportunities to earn additional income are also emerging with the development of the platform economy (EIGE & Eurofound et al., 2023). Indeed, there is increasing flexibility in terms of where, how, and when we work. In particular, the possibility to work from home, using ICT—which was becoming gradually available prior to the COVID-19 pandemic and which expanded tremendously after the outbreak—may make it easier for parents to combine paid work with care and increase the time working adults spend with their families, especially if they perform jobs in distant locations from home (Aksoy et al., 2022; OECD, 2021). In parallel to these developments, legislative efforts have been made across countries to improve working conditions and the compatibility of paid work with private life (European Union, 2019; ILO, 2022). These include regulations reducing the incidence of long working hours, expansion of public childcare, and parental leave entitlements or improvements in employment protection of fixed-term employees.

On the other hand, globalisation, digitalisation, and the shift towards a green economy have also exacerbated sources of uncertainty and have been accompanied by such negative adjustments as salary cuts, job losses, layoffs, bankruptcies, and business failures (Mills & Blossfeld, 2013). Economic uncertainty has become more widespread, with individuals often trapped in precarious or low-quality occupations. Low- and medium-educated workers are affected most strongly by the ongoing changes, as routine jobs are being increasingly taken over by robots or moved to low-income countries (Autor et al., 2006; Goos et al., 2014). Whilst emerging new work arrangements (e.g. on-call work, agency work, crowdworking) offer job opportunities to those who may not otherwise be able to enter employment, they also tend to be unstable, provide insufficient income and social protection, and require complex management of fragmented work schedules (Eurofound, 2017; OECD, 2014). Moreover, the increasing opportunities to work remotely using ICT are making it more difficult to switch off from paid work, which serves to heighten its intensity and blur the boundaries between paid work and private life (Demerouti et al., 2014; Cukrowska-Torzewska et al., 2023). Hence, working in the twenty-first century involves much more uncertainty and requires greater investments of time and effort in workers’ careers, i.e. to care about upgrading one’s skills, combine several jobs, and deal with unpredictable time schedules (Green et al., 2022; Kubicek et al., 2021).

These developments ultimately alter the conditions of earning income and combining paid work and care, which are important determinants of family choices. Young adults must increasingly devote greater time and effort to seeking and sustaining employment, which leaves them with less space for forming intimate relationships, having children, and spending time with family. Furthermore, increased uncertainty leads to the spread of insecurity amongst economic agents and prevents rational decision-making in the family sphere. Hence, individuals may be responding to more than their actual objective economic situation and monetary constraints: narratives of the future, i.e. socially conveyed imagined futures—may play a crucial role in people’s lives (Vignoli et al., 2020a, 2020b). The view of uncertain futures has been further amplified by ongoing climate change concerns, the COVID-19 outbreak of 2020, and the war in Ukraine, all of which are reducing the existential security of Europeans. In her speech on 13 June, 2020, the President of the European Central Bank, Christine Lagarde, described the economic situation as ‘characterised by profound uncertainty’ and stressed that ‘predicting the future has rarely been harder’.

This Special Issue seeks to simulate the debate around the effects of rapid labour market transformations and growing uncertainty on families. It embraces a series of contributions that cover the most recent labour market developments, such as growing employment instability and economic uncertainty (Kreyenfeld et al., 2023; Neels et al., 2024; Scherer & Brini, 2023), financial deterioration (Barbuscia et al., 2023), declining labour market prospects for the low- and middle-educated workers most exposed to automation (Matysiak et al., 2023), declining employment protection in the course of gradual deregulation reforms (Bastianelli et al., 2023), and changing occupational characteristics (Adsera & Querin, 2023). Some of the studies adopt a historical perspective (Neels et al., 2024; Scherer & Brini, 2023; Bastianelli et al., 2023) and investigate how the emerging labour market uncertainties influence partners’ fertility behaviours over longer time spans, encompassing periods of economic busts and booms. The remaining studies concentrate on more recent periods in order to investigate how the most recent changes in labour markets (i.e. robot adoption, the pandemic, and growing time and place flexibility in jobs) are related to family-related behaviours, such as childbearing, and union formation and dissolution (Barbuscia et al., 2023; Matysiak et al., 2023a), as well as gender equality in labour markets (Adsera & Querin, 2023). Three studies adopt a cross-country comparative or even pan-European perspective (Adsera & Querin, 2023; Bastianelli et al., 2023; Matysiak et al., 2023a), whilst others concentrate on such countries as Germany (Kreyenfeld et al., 2023), Italy (Scherer & Brini, 2023), France (Barbuscia et al., 2023), and Belgium (Neels et al., 2024).

In the following section, we review the theoretical approaches and empirical research in the field of labour markets and family life courses to provide a comprehensive background to this Special Issue on Families in the Changing World of Work. We then introduce the articles presented in the issue with a discussion on research gaps and prospects for future research.

## State-of-the-Art on Labour Market and Family Life Courses

### Theoretical Premises

Labour market participation and its conditions have been at the forefront of demographic debate for many decades. As early as the 1950s, Leibenstein (1957) advanced the theory of fertility decline, which presupposes that the decision to have another child depends on household income and the costs of children, which can be divided into direct (i.e. direct expenditures on children) and indirect costs (i.e. foregone earnings due to parents’ limited earning capacities due to care obligations). This theory was further developed by Becker (1993) into a more general theory of New Home Economics (NHE). Within this theoretical model, income from paid work is an important determinant of family formation (including both marriage formation and childbearing) and union stability as long as it is brought by the partner who specialises in market production, whilst the other specialises in household production. According to this theoretical perspective, an increase in women’s labour supply destabilises the role-specialisation model and is therefore responsible for the postponement of marriage formation and fertility, a decline in family size, and an increase in union instability—although Becker allowed for the possibility that higher-income families may have fewer children not only due to the opportunity costs associated with an increase in the female’s income, but also because they may want to invest in the quality of their children.

Another influential perspective on low fertility and the diffusion of new family life courses, which builds, instead, on the sociological foundations of value change and individualisation, is the Second Demographic Transition (SDT; Van de Kaa, 1987; for a global update of the theory, see Lesthaeghe, 2020). At that time, cohabitation was replacing marriage, fertility was being postponed, and more children were being born out of wedlock. The SDT posits that, within postmodern societies, individuals—especially women—emphasise their careers and self-fulfilment more than family and childbearing. In an updated version of his thesis, Lesthaeghe (2010) acknowledged that other components are also important—such as the emancipation of young adults and gender symmetry in daily life—and positively related with fertility recuperation.

Whilst these early theoretical approaches were highly influential, they also received substantial critiques from other scholars. In particular, NHE, which might have been applicable in countries with low levels of female education, was deemed unsuitable in contexts where many highly educated women showed little interest in forming households based on the specialisation principle (Ferber, 2003). In a similar vein, Oppenheimer (1988, 1994, 1997) questioned the idea that specialisation is indeed the most optimal solution for family formation given the rising instability of employment relations. In her theoretical model, Oppenheimer predicted that an increase in women’s education and employment would lead to the postponement of family formation, but may ultimately facilitate union stability as partners are given more time to find a better match. Income pooling, achieved thanks to the participation of both partners in the labour market, may also facilitate fertility decisions and reduce conflict between partners. All in all, the critics of NHE suggest that women’s labour force participation does not have to stand in conflict with family formation and its stability.

Newer approaches, developed in the twenty-first century, underscored the role of institutions and increased male involvement in the family. McDonald (2000, 2013) proposed that low fertility is an outcome of high gender equality in market-oriented institutions—which allow women to educate themselves and work for pay on equal terms with men—and persistently high gender inequality in family-oriented institutions. Esping-Andersen and Billari (2015) developed a multiple equilibria model which presupposed that low fertility is a result of the lack of fit between women’s desire to combine paid work with care on equal terms with their partners and the actual division of paid labour within the couple. Finally, Goldscheider et al. (2015) suggested that low fertility results from an incomplete gender revolution, wherein women’s roles have shifted from full-time carers into earner-and-carers, but men’s have not. All in all, these theoretical approaches envision that fertility can increase with a development of institutions which free women from the round-the-clock provision of childcare and housework and entail the equal division of paid and unpaid labour between partners.

Finally, scholars have underlined the role of economic uncertainty and the subjective perceptions of one’s current job or financial situation. Ranjan (1999) developed a theoretical model which presupposes that fertility postponement is an optimal solution for individuals faced with economic uncertainty given that fertility decisions are irreversible and postponing fertility is possible, at least to a certain extent. Whilst this model may predict the fertility behaviours of couples in which a man’s economic situation is uncertain, the links between the uncertainty around female employment careers and fertility seem less clear. Indeed, according to Kreyenfeld (2010), these depend on whether a woman ‘is expected to be a caregiver or household provider after childbirth’ (Kreyenfeld, 2010: 353). If women’s employment is constrained by normative expectations on men's and women’s social roles, fertility choices shall not be restricted by uncertainties in women’s careers. Likewise, women who plan to continue economic careers after they become mothers would be inclined to postpone fertility until they establish stable positions in the labour market.

The notion of economic uncertainty has been more closely developed in the ‘Narrative Framework’ (Vignoli et al., 2020a, 2020b), which stresses that uncertainty is a forward-looking concept. After all, uncertainty means the absence of clarity about one’s future possibilities, thus hindering the ability to make rational calculations concerning future events (Beckert, 2016; Beckert & Bronk, 2018). In the new context of mounting uncertainty, people tend to consider not only past experiences and present status (the ‘shadow of the past’), but also future expectations, which represent what people expect will happen based on the available information (the ‘shadow of the future’, Bernardi et al., 2019; Huinink & Kohli, 2014). The Narrative Framework introduces the concepts required to operationalise the role of the future in family dynamics; namely, expectations, imaginaries, and narratives of the future. *Expectations* represent an individual’s entire complex belief system about the future state of the world given the expected trends considered. However, the imaginative dialogue that is part of fertility plans also embodies the human capacity to deviate from expectations: personal family *imaginaries* may not coincide with the expected future, but may instead be influenced by a certain ideal or normative orientation around family size or timing. *Personal narratives of the future* synthesise the contingent plan for reaching (or avoiding) desires elicited by the imaginaries.

### Review of the Past Empirical Research

#### Union Formation

In the United States, quantitative research in demography, sociology, and economics has typically shown that (stable) marriage is associated with favourable economic conditions (Carlson et al., 2004; McLaughlin & Lichter, 1997; Smock et al., 1999; Sweeney, 2002; see Sassler & Leichter, 2020 for a review). In Europe, Kohler et al. (2002), and Blossfeld and collaborators (Blossfeld et al., 2005; Blossfeld et al., 2006a, 2006b; Blossfeld & Hofmeister, 2006; Mills & Blossfeld, 2013) were the first to argue that difficulties in finding stable employment for both men and women generate a delay in family formation in favour of a protracted stay in the parental home. Indeed, a number of studies have shown that having a fixed-term or temporary job greatly lowers the chances of forming a long-term union (e.g. Kurz et al., 2005; Noguera et al., 2005; van Wijk et al. (2021). Importantly, a number of studies—both from Europe (e.g. Vignoli et al., 2016) and overseas (e.g. Smock et al., 2005; Evans, 2015)—have suggested that cohabitation, as opposed to marriage, is more suitable given the unpredictability of today’s world. These studies paint an almost unanimous picture: experiences of temporary employment and unemployment—especially amongst men—are associated with a postponement of partnership formation and a preference for cohabitation. Further to these ‘classical’ operationalisations of employment uncertainty, two empirical studies have advanced the importance of conceptualising and measuring economic uncertainty as a future-looking notion. They found that future perspectives are crucial for union formation (e.g. Bolano & Vignoli, 2021; Guetto et al., 2022).

#### Fertility

Empirical macro-level research has suggested a pro-cyclical relationship between economic circumstances and fertility outcomes (Del Bono et al., 2012; Hofmann & Hohmeyer, 2013; Schneider, 2015; Matysiak et al., 2021), although these relationships may have been negative for older generations (Kingsbury & Greenwood, 1992; Rindfuss et al., 2003). However, the interdependencies between economic activity and fertility appear to be more complex at the individual level, where important idiosyncrasies by workers’ gender and socio-economic status, as well as country and time period, are detected.

For men, empirical research has provided evidence consistent with the economic theories of fertility. Namely, it has demonstrated that men’s labour market position (including income) is positively related to fertility (Hart, 2015; Vignoli et al., 2012; Weeden et al., 2006). Similarly, men’s employment instability and men’s unemployment in particular have been shown to be clearly negatively related to fertility (Alderotti et al., 2021).

The findings for women are more diverse, suggesting a more ambiguous relationship, and variations across different welfare state set-ups and over time. Based on 90 estimates of the effect of women’s employment on childbearing, obtained from empirical studies published between 1990 and 2006, Matysiak and Vignoli’s (2008) meta-analysis displayed a negative effect of female employment on childbearing in country groups with relatively poor support for work and family reconciliation and/or traditional gender norms. At the same time, no significant relationship was established in Nordic Europe. More recent studies have also demonstrated a weakening of the negative relationship between women’s employment and birth transitions (Begall, 2013; Zhou & Kan, 2019; Bogusz et al. forthcoming), although the variation in this relationship across country contexts persists: whilst economic activity of women was still found to be negatively related to fertility in Italy (Matysiak & Vignoli, 2013; Rondinelli et al., 2010) and West Germany (Kreyenfeld & Andersson, 2014), similar findings were not obtained for Belgium (Wood & Neels, 2017), Denmark (Andersson et al., 2014), and Norway (Hart, 2015), except in third order births.

The instability of women’s employment has also been found to be significant for fertility. According to Alderotti et al.’s (2021) meta-analysis summarising European research findings from studies published between 1999 and 2018, women in time-limited employment are far more likely to postpone having children than women on unlimited contracts. The effects of women’s unemployment on fertility are, however, more ambiguous (ibid). The meta-study shows that, in the period covered by the original studies, women still clearly used unemployment as an opportunity window for having children in the more traditional settings, though this positive relationship has been weakening over time. More recent evidence for highly educated women (Kreyenfeld, 2010; Kreyenfeld & Andersson, 2014; Pailhe & Solaz, 2012) has consistently suggested that these women plan to continue their occupational careers after childbirth, meaning that their employment stability constitutes an important prerequisite for their childbearing (Kreyenfeld, 2010; Matysiak, 2009; Wood & Neels, 2017). On the whole, these findings suggest that women’s economic roles have been changing and that the stability of females’ careers plays an increasingly important role for fertility behaviours.

Empirical research has also increasingly supported the ‘Narrative Framework’ perspective, with very little gender differences. Net of more ‘conventional’ aspects that have been emphasised in earlier research on family dynamics such as present and past economic circumstances, the importance of future narratives has been supported by analyses based on primary experimental data for fertility intentions (Guetto et al., 2022; Vignoli et al., 2022; Matera et al., 2023), and secondary data for fertility intentions (e.g. Gatta et al., 2022; Lappegård et al., 2022; Manning et al., 2022) and fertility behaviours (e.g. Brauner-Otto & Geist, 2018; Guetto et al., 2023a).

#### Union Dissolution

The relationships between men’s employment and divorce seem to be more complex than those of men’s employment and fertility. On one side, the *relational stress hypothesis* (Conger et al., 1990; Liker & Elder, 1983) suggests that the male partner’s unemployment or employment instability increases psychological distress and exacerbates marital discord, thus heightening the risk of divorce. Conversely, the *cost of divorce hypothesis* (Cherlin, 1979) argues that unfavourable economic conditions may reduce divorce by rising its relative cost, i.e. legal settlements, household relocation, or increasing the costs of consumer durables (Amato & Beattie, 2010; Cohen, 2014; Fischer & Liefbroer, 2006). In line with the relational stress hypothesis, prior research has shown that men’s unemployment has been repeatedly linked to union dissolution (Conger et al., 1990; Doiron & Mendolia, 2012; Hansen, 2005; Liker & Elder, 1983; Ruggles, 1997; Starkey, 1996; Poortman & Mills, 2012).

Likewise, theoretical ambivalence exists for the direction of the effects of women’s employment on divorce. The NHE predicts that women’s employment increases divorce risk as it jeopardises the specialisation of partners in paid and unpaid labour. The positive effects of women’s employment on divorce can be also predicted on the basis of the *independence hypothesis*, which states that employment gives women the resources to terminate unhappy marriages (Hobson, 1990; Ruggles, 1997; Schoen et al., 2002). On the other hand, women’s employment can be an additional source of income, which improves the couple’s financial situation and weakens economic strains (Oppenheimer, 1997). Past studies have found the relationship between woman’s employment and divorce to vary across countries from negative to positive, with negative effects most often reported in Scandinavian countries (Hansen, 2005; Jalovaara, 2003; Cooke et al., 2013; Vignoli et al., 2018; see also Härkönen & Dronkers, 2006 and Matysiak et al., 2014 for a similar discussion on educational gradient and marital stability). Other studies have demonstrated that the negative relationship between female employment and divorce substantially weakens or even disappears amongst couples with more equal divisions of household labour (Mencarini & Vignoli, 2018; Sigle-Rushton, 2010), which is consistent with gender equality theories (Esping-Anderssen & Billari, 2015; Goldscheider et al., 2015).

The majority of the empirical evidence on the topic does not account for time-limited jobs. Nonetheless, time-limited work has been proven to contribute to increased work–family conflict and relationship strife (Scherer, 2009; Steiber, 2009), as well as decreased relationship satisfaction (Blom et al., 2020) which, as with unemployment, may serve to increase the risk of dissolution. Recently, Bastianelli and Vignoli (2022) suggested that the effect of employment instability on union dissolution is gender-specific in Italy: joblessness and limited-time employment facilitate men’s dissolution, whilst, for women, joblessness is an inhibitor for dissolution, and time-limited and permanent employment do not substantially differ.

## Special Issue Outline and Contribution

Despite the rich empirical research in the field of labour markets and family formation, many aspects remain unresolved. The papers presented in this Special Issue substantially contribute to filling some of these research gaps. First, there has been a notable lack of research on the changes in the relationship between labour force participation and family-related behaviours. Many previous studies have relied on data from single countries observed in confined periods of time which do not allow for comparisons across time. Accordingly, it has not been possible to investigate whether certain relationships have weakened or amplified in magnitude, if the role of unstable employment on family formation has intensified, or whether the role of women’s employment on fertility has become positive.

Scherer and Brini (2023) seek to fill this gap by demonstrating changes in the relationship between employment instability and fertility over a 20-year period (2000–2020) in Italy. Using data from the Italian Labour Force Survey, the authors confirmed past findings about the negative effects of employment instability on first and second births (also for women), but not on third births. It should be noted that they demonstrate that these effects have definitely amplified, in particular for women. They also find the negative effects of unemployment on first and second births to be particularly strong for highly educated women and men. Whilst the findings of Scherer and Brini (2023) are consistent with prior research on Italy regarding women (Guetto et al., 2023b; Vignoli et al., 2020), the pronounced negative effect amongst highly educated men represents a novelty in line with past research for Germany (Kreyenfeld, 2010) and France (Pailhe & Solaz, 2012).

Neels et al. (2024) also trace fertility developments across longer time periods. The authors use longitudinal microdata from Belgian population censuses, covering a period of 50 years (1960–2010). They confirm the findings of Ní Bhrolcháin and Beaujouan (2012) that educational expansion was a crucial driver of fertility postponement, but also demonstrate that postponement accelerates during an economic recession, only to decelerate after. However, the authors find no intensification of the positive effect of economic recessions on fertility postponement over time. Neels et al. (2024) also make another important contribution to the field (and demographic research in general) by using micro-simulation tools to assess the role of economic recessions not only for individual fertility behaviours, but also on aggregate fertility. This is an important step forward in fertility research, as the majority of previous studies have indeed investigated how changing economic circumstances affect the fertility behaviours of individuals, but have neglected to provide information on how the effects translate into aggregate fertility, which is often of interest to policy makers.

Bastianelli et al. (2023) also adopt a longer time perspective and make yet another contribution, namely by examining the role of the changes in labour market policies, and more specifically in employment protection legislation, on fertility. Past studies in family demography have so far largely focused on the role of family or reconciliation policies. Changes in employment protection legislation have rarely been mentioned in the demographic literature (for exceptions, see Adsera, 2004, 2005; Rovny, 2011; Matysiak & Węziak-Białowolska, 2016). Adopting a macro-level perspective and covering the years 1990–2019 in 19 European countries, the authors find that an increase in employment protection positively affects the fertility of regular workers, but this positive effect substantially weakens or even turns negative with an increase in labour market dualism, measured by a gap in employment protection of permanent versus temporary workers. Not only does this study provide novel findings to demographic research, but it also adds to the literature on the social consequences of deregulation reforms in that reductions to employment protection for temporary, rather than permanent, workers create difficulties for young adults to enter stable employment and lead to an explosion in temporary contracts (Barbieri & Cutuli, 2016; Gebel & Giesecke, 2016). Bastianelli et al. (2023) demonstrate that deregulation reforms not only increase labour market dualism but also reduce fertility.

The difficulties with disentangling tempo from quantum effects is another limitation of past research in the field of family and labour markets (and demographic research in general). Consequently, it remains unclear whether individuals who face difficulties with entering (stable) employment only postpone having a child or forego it definitely. Kreyenfeld et al. (2023) overcome this difficulty by implementing mixture cure models. Their aim is to examine the role of social class in fertility behaviour, finding that, net of timing effects, members of higher social classes are more likely to have a second child than members of the lower social classes in Germany. In other words, regardless of the shifts in birth timing, both women and men in upper social classes are more likely to have two children than their lower-class counterparts, in particular the skilled and semi-/unskilled. They also report that women who were not employed at first birth were clearly less likely to have a second child compared to those who were employed. The study concludes that the upper social classes have an economic advantage, allowing their members to enlarge their families. It also demonstrates an important change in the economic position of women in the household in Germany, which for a long-time pursued family policies directed at maintaining the traditional division of labour between women and men (see Bogusz et al. forthcoming for a similar finding).

Three studies contribute to the literature by examining the consequences of labour market changes caused by specific and relatively recent developments, namely changes in the structure of labour demand caused by the digital revolution (Adsera & Querin, 2023; Matysiak et al., 2023a) and an increase in financial difficulties due to COVID-19 (Barbuscia et al., 2023). The expansion of new technologies, such as those which facilitate remote communication (e.g. ICT) or replace human labour (e.g. industrial robots or automats), has been profoundly transforming the labour markets over the last three decades. Not only do these technologies change the ways in which we work, such as allowing for more flexible working or working from distant locations, but also determine who is in more demand within the labour market. Economic research has demonstrated an increase in the labour demand for cognitive workers, especially those who display strong analytical and social skills, and a decline in the demand for workers who perform routine tasks at work (Acemoglu & Autor, 2011; Autor et al., 2006; Goos et al., 2014; Hardy et al., 2018). In such a context, Adsera and Querin (2023) explore how these changes in the labour demand may affect men and women’s earnings. The authors demonstrate that women more often work in jobs which are less intense in machine-use and require less autonomy in decision-making, but are high in social contact. Such jobs are often less paid, placing women at a relatively disadvantaged position in terms of future-earning prospects in comparison with men (see also Matysiak et al., 2023b). The fertility consequences of the changes in the structure of the labour demand driven by implementation of industrial robots (automation) were, in turn, addressed by Matysiak et al. (2023a). The authors argue that the introduction of industrial robots causes structural changes in the labour market which permanently change the demand for labour, putting low- and middle-skilled workers at a disadvantage and benefitting the highly skilled. They consistently find some traces of negative fertility effects of automation in Germany and Italy (though not France or the UK), especially in regions with less-educated populations and large manufacturing sectors in the early stages of the digital revolution. Their findings align with other studies that have addressed the fertility consequences of structural labour market changes (Anelli et al., 2021; Seltzer, 2019; Bogusz et al. forthcoming), which is an emerging topic in family demography.

Finally, Barbuscia et al (2023) examine the effects of the economic consequences of the COVID-19 pandemic on union stability. The COVID-19 pandemic, and the responses to it, escalated the uncertainty at the core of the public debate and personal lives. People began to feel insecure in their daily lives due to the risk of contagion and the possibility of losing one’s job and/or having a reduced standard of living. This additional uncertainty condition can be seen as an additional consequence of globalisation, given that the swift spread of the pandemic is intricately linked to extensive exchanges and global interdependencies. Using data from the nationally representative EPICOV survey, which followed individuals throughout the first year of the pandemic in France, Barbuscia et al. (2023) examined separation rates and their association with different measures of employment and income uncertainty, including both pre-pandemic conditions, and changes occurring during and after the first lockdown in the spring of 2020 in France. Their results show increased rates of separation, especially amongst younger people, during the six months following the first lockdown, and a return to rates more similar to those observed in usual times afterwards. Individuals who were unemployed and had lower incomes before the outbreak were more likely to separate soon after the lockdown, whilst changes in employment conditions due to the lockdown were not linked with a higher separation risk. Importantly, self-declared deterioration in financial conditions, especially when declared by men, was associated with higher separation risk for the entire observation year.

Overall, the articles collected in this Special Issue clearly highlight how labour force participation and its conditions determine family life courses. The feeling of economic uncertainty, the threat of unemployment or precarious employment, or financial difficulties can lead fertility to being postponed and the risk of union disruption to increase. These effects intensified where labour market deregulation went hand in hand with labour market dualization and were more pronounced during the periods of economic hardship, such as economic recessions or the COVID-19 pandemic (Scherer & Brini, 2023; Neels et al., 2024; Matysiak et al., 2023a; Barbuscia et al., 2023; Bastianelli et al., 2023). It also appears that the effects of economic activity on family-related behaviours have become less gendered as women increasingly gain economic independence and young adults face difficulties with finding and maintaining employment (Scherer & Brini, 2023; Kreyenfeld et al., 2023). This observation casts doubt on the validity of the NHE for explaining the links between labour force participation and family-related behaviours in contemporary European societies. Instead, it grants credibility to the arguments of Oppenheimer (1997), who predicted that women’s economic activity will play an increasingly positive role in the process of family formation as employment becomes less stable and taken for granted. Finally, it would seem that highly educated workers and members of the upper social classes face increasingly better conditions for realising their fertility intentions due to their having more favourable economic resources and wider opportunities for earning income than the low- and middle-skilled workers who are more exposed to unstable and fragmented employment and low pay. This economic advantage is seemingly reflected in the fertility behaviours of young adults (Kreyenfeld et al., 2023; Matysiak et al., 2023a). For the time being, completed fertility amongst highly educated women who reached the end of their reproductive spans is still lower than amongst women with low and middle education (Nisen et al., 2021). Furthermore, time will tell whether the transformations in labour markets will bring a reversal in the educational gradient in the completed fertility of younger cohorts.

## Prospects for Research

Although the articles included in this Special Issue provide new additional insights into the field of the interdependencies between labour force participation and family dynamics, numerous open questions remain to be addressed. Indeed, future research should more closely examine how the dramatic transformations in the labour market, caused by digitalisation, affect family formation and its stability. These changes have been occurring in Europe for at least three decades and have led to long-lasting changes in the structure of labour demand, which shifted away from middle-skilled workers performing routine tasks (OECD, 2019; World Bank, 2018). The latest developments in artificial intelligence may further contribute to these changes, but this time also affecting cognitive workers (OECD, 2023). Demographic research has made a significant step toward understanding how short-term changes in labour market conditions, caused by cyclical swings in the economy, affect family-related behaviours (e.g. Comolli, 2017; Matysiak et al., 2021; Neels et al., 2013; Schneider, 2015). However, comparatively little is known about the demographic effects of the long-term structural changes in the economy brought about by substantial technological transformations, such as the current digital revolution. Whilst cyclical swings in the economy lead to postponement of family formation and its recuperation in the aftermath of economic crises, structural changes to the labour market, which are more enduring, may not only affect the timing of family-related events, but also their occurrence, in particular amongst the low- and middle-educated workers who seem to be most negatively affected by the ongoing changes. More effort should thus be invested in developing methods and approaches which allow one to distinguish between the effects of the ongoing labour market changes on the quantity and timing of fertility.

Little is also known on how the new forms of work (e.g. working from home or platform work) affect family-related behaviours. For instance, does social isolation related to working from home affect union formation? Are people who work from home more likely to have children as they are better able to achieve a work–life balance? Or does the blurring of the boundaries between paid work and family life coupled with high work intensity, which are often consequences of working from home, reduce partners’ desire to have a child? Only a handful of studies have examined the role of working from home for fertility and reached conflicting findings. For instance, whilst Sinyavskaya and Billingsley (2015) showed that women able to work from home had higher first and second birth intentions, Osiewalska et al. (2024) found such a positive relationship between this mode of work and first and second births only amongst women who would otherwise face long commuting times, and Kurowska et al. (2023) reported a negative relationship between working from home and fertility intentions during the COVID-19 pandemic. We know even less about fertility and the emerging opportunities for paid work in the platform economy. This mode of work provides workers with little social and employment security, which likely does not facilitate family formation, but, on the other hand, provides flexibility which may help work and family reconciliation (Lehdonvirta, 2018; EIGE & Eurofound, 2023).

Future research should also integrate conventional economic and labour market models and measurement approaches to the study of families by acknowledging subjective assessments and future narratives to gain a more compelling and up-to-date view of family dynamics in the changing world of work. One theoretical and one methodological consideration are pertinent on this matter. From a theoretical perspective, the simultaneous consideration of both the objective and subjective sides of economic uncertainty complicates matters, but also serves to strengthen scholarship on family dynamics. Relying solely on objective indicators of employment-related difficulties provides a narrow perspective of family dynamics: the specter of the future also matters. A fruitful avenue for studying family life courses amidst uncertainty involves incorporating prospective measures of uncertainty into empirical analyses. The potential interaction between objective and subjective measures of uncertainty is also worth exploiting—namely, we suggest exploring how subjective appraisals of employment uncertainty affect family dynamics across different objective employment conditions. We contend that this forward-looking perspective is especially relevant and urgent in a world grappling with pervasive global uncertainties. From a methodological perspective, existing studies have typically explored the effects of indicators of labour market circumstances and employment uncertainty on family-related transitions within a time-to-event framework, which makes it difficult to interpret the results in causal terms. As recently re-emphasised by Kreyenfeld (2021), the association between time-varying indicators of employment status and family-related events may be influenced by anticipation effects. For instance, a woman’s change from employment to non-employment may be induced by the intention to enter a union, have a child, or dissolve an unhappy union. Considering narratives of the future thus represents a possible solution for tackling anticipation and integrating people’s future plans into the statistical models (Kreyenfeld, 2021).

Finally, although this Special Issue focuses on the changing world of work, there is a need to expand the discussion around the role of uncertain futures from economic and employment uncertainty to other forms of uncertainty. The challenges ahead extend beyond economic unpredictability and shifts in the labour market. A broader spectrum of uncertainties is gradually seeping into the social fabric of contemporary nations. Examining how new forms of uncertainty related to the environment, societal dynamics, and political landscapes influence and shape family life courses presents a novel and promising avenue for future research.
